# Exercise Habits, Including Exercising With Partners, and the Prevalence of Self-Reported Constipation in Young Japanese People: A Cross-Sectional Study

**DOI:** 10.7759/cureus.74455

**Published:** 2024-11-25

**Authors:** Junichi Watanabe, Shinya Furukawa, Yasunori Yamamoto, Aki Kato, Katsunori Kusumoto, Eiji Takeshita, Yoshio Ikeda, Naofumi Yamamoto, Yuka Saeki, Teruki Miyake, Osamu Yoshida, Yoichi Hiasa

**Affiliations:** 1 Department of Physical Therapy, Faculty of Health Sciences, Yamagata Prefectural University of Health Sciences, Yamagata, JPN; 2 Health Services Center, Ehime University, Matsuyama, JPN; 3 Endoscopy Center, Ehime University Hospital, Toon, JPN; 4 Faculty of Collaborative Regional Innovation, Ehime University, Matsuyama, JPN; 5 Gastroenterology and Metabology, Ehime University Graduate School of Medicine, Toon, JPN

**Keywords:** exercise habits, exercise partners, functional constipation, japanese young people, physical activity

## Abstract

Background: Constipation is a very common medical issue among the general population worldwide. However, the association between exercise habits and constipation is still not fully understood. Additionally, no evidence regarding the association between exercise partners and constipation exists. This study aimed to evaluate this issue in a young Japanese population, taking the presence or absence of an exercise partner as an additional variable.

Methods: The study subjects consisted of 12,497 Japanese university students. Information on constipation, exercise frequency, exercise intensity, and exercise partners was obtained through a self-administered questionnaire. Constipation was defined as present if a student answered “Yes” to the question, “Have you been constipated often recently?”

Results: The prevalence of self-reported constipation was 6.5%. Frequency and intensity of exercise were independently inversely associated with constipation. After adjustment for age, body mass index, drinking, smoking, anemia, and sports injury, exercise with groups and friends was independently inversely associated with constipation (groups: adjusted odds ratio (OR) - 0.70 (95% confidence interval (CI): 0.53-0.90), friends: adjusted OR - 0.56 (95% CI: 0.42-0.74)). After further adjustment by adding intensity and frequency of exercise to confounding factors, only the association between exercise with friends and constipation was still significant (adjusted OR: 0.61 (95% CI: 0.39-0.96)).

Conclusions: In this young Japanese population, the frequency and the intensity of exercise and the presence of exercise partners might be independently inversely associated with self-reported constipation. Exercising, especially exercising with others, may have a preventive effect on constipation, and opportunities to exercise with others should be provided.

## Introduction

Constipation is a common gastrointestinal disorder among general humans worldwide. A previous report indicates that the prevalence of constipation ranges from 2.6% to 26.9% [1]. It is estimated that 70-90% of patients with constipation report bothersome symptoms such as bloating, straining, and hard stool persisting for many years, with a negative impact on health-related quality of life [2,3]. Increased physical activity (PA) has long been recommended as part of the management of constipation.

Several studies have shown an inverse association between exercise and constipation. An Icelandic, a Swedish, a Hong Kong, two Chinese, and three Japanese studies showed inverse associations between constipation and PA [4-11]. On the other hand, no association between PA and constipation was found in a study from the United States, a study from the United Kingdom, and two Spanish studies [12-15].

Exercising with others may have a stronger beneficial effect on health compared to exercising alone [16-20]. However, no study, to the best of our knowledge, has been done to investigate the association between exercise partners and constipation. Having an exercise partner increases PA, and even after adjustment for PA, exercise with others still has a positive effect on health [17,18]. The underlying mechanism of the association between exercising with others and health is still unclear. To accumulate further evidence, we aimed to evaluate the association between self-reported exercise habits, including exercising with a partner, and self-reported constipation in a young Japanese population.

## Materials and methods

Study population

Students who were encouraged to undergo a health check-up every year by email and noticeboards were included in this cohort group. All university health checkup recipients were eligible. In this study, we enrolled 12,523 students who had health examinations at Ehime University (Ehime, Japan) between April 2015 and April 2017. A specific questionnaire pertaining to constipation was sent to all subjects at the time of their health checkup. We excluded those with missing data in their copies of the questionnaire. After 26 subjects were excluded due to incomplete data, the final analysis sample in this study consisted of 12,497 subjects, all of whom were assessed for constipation and exercise habits.

Ethics statement

All subjects were provided an opt-out, and the study protocol was developed in accordance with the ethical guidelines of the Declaration of Helsinki. This study was approved by the ethics committee of the Ehime University Graduate School of Medicine (approval no. 1610012).

Measurements

Information on smoking, drinking, and medical history was collected using a self-administered questionnaire. Current smoking was defined as positive if a study subject reported smoking. Current drinking was defined as positive if a study subject reported consuming alcoholic beverages, regardless of frequency. Height was measured to the nearest millimeter using a stadiometer, with the subject standing completely erect. Weight was measured with the subject wearing light clothing. The body mass index (BMI) was calculated as the weight in kilograms divided by the height in meters squared.

Assessment of exercise habits, including the presence/absence of exercise partners

We used a self-administered questionnaire to estimate exercise habits using the following questions. (1) Frequency of exercise: “How frequently do you exercise?” (none, one-to-two times per month, one-to-three times per week, and four or more times per week); (2) intensity of exercise: “What is your main type of exercise?” (light exercise (normal walking, housework, vehicles, ball playing, stretching, etc.), moderate exercise (fast walking, stairlifts, bicycle, softball, hiking, golf, radio calisthenics, etc.), and intense exercise (jogging, climbing stairs, badminton, tennis, mountain climbing, swimming, muscle training, judo, etc.)); and (3) exercise partner: “Who do you mostly exercise with?” (group, friends, and alone).

Assessment of constipation

We used a self-administered questionnaire to estimate constipation. Self-reported constipation was defined as present if the student answered “Yes” to the question, “Have you been constipated often recently?”

Statistical analysis

The sample size was calculated based on previous data regarding exercise and constipation. A previous study showed that the prevalence of constipation in the exercise group (n=4,282) and the non-exercise group (n=485) were 3.01% and 5.98%, respectively [5]. Precisely 763 subjects were needed to provide 80% power and a significance level of 5% to show a significant difference between the exercise group and the non-exercise group. The frequency of exercise was divided into four categories: none (reference), low (one to two times per month), moderate (one to three times per week), and high (four or more times per week). The intensity of exercise was divided into four categories: none (reference), light exercise, moderate exercise, and high-intensity exercise. The four categories of exercise partners were as follows: none (reference), with a group, with friends, and alone. Estimations of crude odds ratios (ORs) and their 95% confidence intervals (CIs) for self-reported constipation in relation to exercise frequency, exercise intensity, and exercise partners were performed using a logistic regression analysis. Multiple logistic regression analyses were used to adjust for potential confounding factors. Age, sex, BMI, drinking, smoking, anemia, and sports injury were selected as potential confounding factors. Statistical analyses were mainly performed using the SAS software package version 9.4 (SAS Institute Inc., Cary, North Carolina, United States). All probability values for statistical tests were two-tailed, and p<0.05 was considered statistically significant.

## Results

Subject characteristics

Table 1 shows the characteristics of the 12,497 study participants. The percentage of men was 60.4% in this cohort. The mean age and BMI were 20.1 years and 21.37, respectively. The percentages of smoking and drinking were 5.4 % and 10.0 %, respectively. The prevalence of self-reported constipation was 6.5% in this cohort.

**Table 1:** Clinical characteristics of 12,497 study participants

Variable	Total (N=12,497)
Age, years, mean ± SD, (range)	20.1 ± 3.1, (18-63)
Sex (Male/Female)	7546/4951
Body mass index, mean ± SD	21.37 ± 3.08
Smoking, n (%)	669 (5.4)
Drinking, n (%)	1253 (10.0)
Medical history	-
Heart murmur, n (%)	70 (0.6)
ECG abnormality, n (%)	112 (0.9)
Anemia, n (%)	370 (3.0)
Traffic accident, n (%)	167 (1.3)
Sports injury, n (%)	424 (3.4)
Exercise habit, n (%)	-
Frequency of exercise habit	-
None, n (%)	3940 (31.5)
One to two times/month, %	3704 (29.6)
One to three times/week, %	3077 (24.6)
Four or more times/week, %	1776 (14.2)
Intensity of exercise	-
None, %	3940 (31.5)
Light exercise, n(%)	2130 (17.0)
Moderate exercise, n(%)	3248 (26.0)
Intense exercise, n(%)	3179 (25.4)
Exercise with	659 (5.3)
None, n(%)	3940 (31.5)
Groups, n(%)	2242 (17.9)
Friends, n(%)	2040 (16.3)
Alone, n(%)	4275 (34.2)
Self-reported constipation, n (%)	659 (6.5)

Table 2 shows the crude and adjusted ORs and 95% CIs for self-reported constipation in relation to exercise habits. The prevalence of self-reported constipation among subjects with none, low, moderate, and high frequency of exercise were 7.3%, 4.5%, 4.2%, and 4.2%, respectively. After adjustment for age, sex, BMI, drinking, smoking, anemia, and sports injury, low, moderate, and high frequency of exercise were independently inversely associated with self-reported constipation (adjusted ORs were for low: 0.77 (95% CI: 0.63-0.94), for moderate: 0.75 (95% CI: 0.60-0.94), and for high: 0.70 (95% CI 0.53-0.91), and the p-value for the trend was p=0.002). The prevalence of self-reported constipation for low, moderate, and high-intensity exercise was 5.4%, 4. 7%, and 3.2%, respectively. Low, moderate, and high-intensity exercise were associated with self-reported constipation after adjustment for confounding factors (adjusted ORs were for low: 0.77 (95% CI: 0.62-0.97), for moderate: 0.77 (95% CI: 0.63-0.95), and for high: 0.68 (95% CI: 0.53-0.87), and the p-value for trend was p=0.001).

**Table 2:** Crude and adjusted odds ratios (ORs) and 95% confidence intervals (CIs) for self-reported constipation in relation to exercise habits # p<0.05 was considered statistically significant. Multiple logistic regression analyses were used to adjust for potential confounding factors. Odds ratios were adjusted for age, sex, body mass index, drinking, smoking, anemia, and sports injury.

Variable	Prevalence, n (%)	Crude OR (95% CI)	Adjusted OR (95% CI)
Self-reported constipation	-	-	-
Frequency of exercise habit	-	-	-
None	289/3940 (7.3)	1.00	1.00
One to two times/month	167/3704 (4.5)	0.60 (0.49–0.73) ^#^	0.77 (0.63–0.94) ^#^
One to three times/week	128/3077 (4.2)	0.55 (0.44–0.68)^#^	0.75 (0.60–0.94) ^#^
Four or more times/week	75/1776 (4.2)	0.56 (0.43–0.72) ^#^	0.70 (0.53–0.91) ^#^
p-value for trend	-	-	0.002
Intensity of exercise	-	-	-
None	289/3940 (7.3)	1.00	1.00
Light exercise	115/2130 (5.4)	0.72 (0.58–0.90) ^#^	0.77 (0.62–0.97) ^#^
Moderate exercise	153/3248 (4.7)	0.63 (0.51–0.76) ^#^	0.77 (0.63–0.95) ^#^
Intense exercise	102/3179 (3.2)	0.42 (0.33–0.53) ^#^	0.68 (0.53–0.87) ^#^
p-value for trend	-	-	0.001

Table 3 shows that the prevalence of self-reported constipation in exercise with groups, with friends, and alone was 3.8%, 3.0%, and 5.3%, respectively. After adjustment for age, BMI, drinking, smoking, anemia, and sports injury (model 1), exercising with groups and exercising with friends were independently inversely associated with self-reported constipation (adjusted OR with groups: 0.70 (95% CI: 0.53-0.90) and with friends: 0.56 (95% CI: 0.42-0.74)). The association between exercising alone and constipation was not significant. After further adjustment by adding intensity and frequency of exercise to the list of confounding factors (model 2), only exercising with friends was independently and positively associated with self-reported constipation (adjusted OR: 0.61 (95% CI: 0.39-0.96)).

**Table 3:** Crude and adjusted odds ratios (ORs) and 95% confidence intervals (CIs) for self-reported constipation in relation to exercise partner. # p<0.05 was considered statistically significant. Multiple logistic regression analyses were used to adjust for potential confounding factors. *Model 1: Odds ratios were adjusted for age, sex, body mass index, drinking, smoking, anemia, and sports injury. **Model 2: Odds ratios were adjusted for age, sex, body mass index, drinking, smoking, anemia, sports injury, exercise frequency, and exercise intensity.

Variable	Prevalence, n (%)	Crude OR (95% CI)	Adjusted OR (95% CI)*	Adjusted OR (95% CI)**
Self-reported constipation	-	-	-	-
Exercise with	-	-	-	-
None	289/3940(7.3)	1.00	1.00	1.00
Groups	84/2242 (3.8)	0.49 (0.38–0.63) ^#^	0.70 (0.53–0.90) ^#^	0.78 (0.46–1.33)
Friends	61/2040 (3.0)	0.39 (0.29–0.51) ^#^	0.56 (0.42–0.74) ^#^	0.61 (0.39–0.96) ^#^
Alone	225/4275 (5.3)	0.70 (0.59–0.84) ^#^	0.84 (0.70–1.02)	0.95 (0.64–1.38)

## Discussion

In the present study, frequency and intensity of exercise and exercise partners were independently inversely associated with self-reported constipation in a young Japanese population. To the best of our knowledge, this is the first study to show an inverse association between exercise partners and constipation in a young Japanese population.

Several studies reported the association between exercise and constipation (Appendix 1). However, the association between exercise habits and constipation was still inconsistent. Regular exercise was significantly inversely associated with constipation in two Chinese studies [6,7] and two Japanese cohort studies [9,11]. In a Swedish study of 15,010 participants, leisure physical activity was significantly inversely associated with constipation [4]. Similarly, in adolescents, exercise habit was inversely associated with constipation in an Icelandic case-control study [5], a Hong Kong study [8], and a Japanese study [10]. On the other hand, no association between exercise habits and constipation was found in a study from the United States of 1,069 participants aged 24-77 years [12], a study from the United Kingdom of 94 primiparous pregnant women aged 19-40 years [13], a Spanish study of 414 participants aged over 50 years [14], and another Spanish study of 415 participants aged 43.8 years [15].

Some studies showed an association between the frequency and the intensity of exercise and constipation. The Icelandic study defined regular exercise as at least three times a week and long enough to make one sweat [5]. The Japanese study defined good (23 or more metabolic equivalents (METs) hours/week) or poor (less than 23 METs hours/week) [11], and a British study defined intense activity (fast cycling, swimming, and aerobics), moderate activity (cycling or swimming at a normal pace), or light activity (walking or meditation) [13]. Previous studies showed that the prevalence of constipation in African and White people was high. Race might affect the prevalence of constipation [21,22]. The discrepancies among these study results may be explained, at least in part, by the differences in the definition of constipation, exercise intensity, race, sample size, study design, assessment of exercise, and age.

Several studies showed the relationship between exercising with others and chronic diseases. Exercising with others was others was beneficial for depression [16], mental status [17], and subjective health status [18]. Additionally, exercising with others was significantly inversely associated with functional dyspepsia [19] and irritable bowel syndrome [20]. The findings in this study are consistent with the previous evidence regarding exercising with others and diseases.

The underlying mechanism linking exercise and constipation remains unclear. In particular, the mechanism underlying the beneficial effect of exercising with others remains poorly understood. Exercise may enhance intestinal gas clearance [23] and promote bowel movements [24]. In addition, exercise stimulates appetite, and sufficient dietary intake leads to a higher frequency of bowel movements. Having an exercise partner increases PA, and even after adjustment for PA, exercising with others still has a positive effect on health [17,18]. Previous research has shown a positive correlation between depression and constipation and has clarified the causal relationship between depression and constipation [25]. Exercising with friends and groups might improve constipation by reducing depressive symptoms. Exercising with others may help reduce symptoms of depression. Even for the young generation, social relationships might be one mechanism underlying the effects of exercising with others for constipation [19]. Additionally, we may not collect the residual confounding factors to adjust for the exercise partner and constipation. Further study on the mechanism, especially the association between exercising with others and constipation, is needed in the future.

Several limitations of the present study warrant mention. First, as this study is cross-sectional, a causal relationship between exercise and constipation cannot be proven. Second, the information regarding participants’ exercise habits and the presence of exercise partners was obtained using a self-administered questionnaire in the present study. Third, constipation was defined by self-report, not using a validated questionnaire. Fourth, the detailed dietary habits/history of the participants was not assessed. Finally, the patients in the current study might not be representative of the young Japanese population. Given that the present cohort consisted only of university students, it is possible that the relatively high educational status of our population affected health behaviors. A Japanese internet study randomly selected 3000 people from a general consumer panel monitor to match the population composition ratio by prefecture, gender, and age group and reported that the prevalence of self-reported constipation was 51.5% [26].

## Conclusions

In the young Japanese population, the frequency and intensity of exercise and the presence of exercise partners might be independently inversely associated with self-reported constipation. Exercising, especially exercising with others, may have a preventive effect on constipation, and opportunities to exercise with others should be provided. We acknowledge the need to verify our results by means of additional prospective studies in Asian countries.

## Appendices

**Table 4:** The association between exercise and constipation in previous studies METs: metabolic equivalents EpiHealth questionnaire: Lund University, Malmö, Sweden; Kaiser Physical Activity Survey (KPAS): Ainsworth BE, Sternfeld B, Richardson MT, Jackson K (1999); APA PsycTests; https://doi.org/10.1037/t23316-000

Reference no.	Authors	Title	Year	Country	Subject	Design	Definitions of exercise	Definition of constipation	Results
4	Seidenfaden et al.	Physical activity may decrease the likelihood of children developing constipation	2018	Iceland	190 children (constipation group=60, control group=130), aged 1 to 18 years	Case-control study	Physical activity (<3 times per week or >3 times per week, １hour each time ), sedentary lifestyle (How long does your child sit in front of the television or the computer each day? (1) Less than 30 minutes, (2) 30-60 minutes, (3) 1-2 hours, (4) 2-3 hours, (5) 3-4 hours, (6) More than 4 hours).	Diagnosed with constipation by a doctor	Physical activity may affect the likelihood of developing constipation in older children.
5	Ohlsson and Manjer	Physical inactivity during leisure time and irregular meals are associated with functional gastrointestinal complaints in middle-aged and elderly subjects	2016	Sweden	The cohort included 16,840 subjects between 45 and 75 years of age.	Multicenter longitudinal cohort	Leisure time physical activity: (1) mostly sitting, (2) light activity, (3) walking 30 minutes/day, (4) activity for 30–60 minutes/day, (5) strenuous activity for 60 minutes/day.	EpiHealth questionnaire	The higher the degree of physical activity, the lower the risk for all kinds of gastrointestinal complaints.
6	Huang et al.	Prevalence and risk factors of chronic constipation among women aged 50 years and older in Shanghai, China	2017	China	1,950 women aged 50 years and older	Cross-sectional survey	Regular physical exercise was defined as participation in any physical activity that was performed long enough to sweat at least 3 times a week.	Rome III criteria	Multiple logistic analyses showed that less physical exercise was a less significant risk factor for chronic constipation in the population of women aged 50 years and older.
7	Shi et al.	Epidemiology and risk factors of functional constipation in pregnant women	2015	China	Data were collected from 1,698 pregnant women with a mean age of 28.12±2.80 years and an age range of 18 to 45 years.	Cross-sectional survey	With or without exercise.	Diagnosed using the Rome III criteria	Multivariate logistic regression analysis showed that the prevalence of functional constipation in pregnant women is related to exercise.
8	Huang et al.	Physical activity and constipation in Hong Kong adolescents	2014	Hong Kong	A total of 33,692 Form 1–7 students (44.9% boys, mean age: 14.8 years, SD: 1.9 years)	Cross-sectional survey	Based on the recommendation of at least 60 minutes of physical activity per day, those who chose one hour or longer durations were considered to have sufficient exercise (reference group) and otherwise as insufficient exercise.	Constipation was defined as once every three days or less frequently, consistent with one of the symptoms in Rome III criteria of ‘‘fewer than three defecations per week’’ and the definition used in previous studies.	Constipation was associated with insufficient physical activity and excessive sedentary behaviors among Chinese adolescents with a dose-response relation.
9	Otani et al.	Prevalence and risk factors of functional constipation in the Rome IV criteria during a medical check-up in Japan	2021	Japan	N=10,658, age (years, median (IQR)): 51.0 (43.0–60.0)	Cross-sectional survey	Exercise for ≥30 minutes at least twice a week for ≥1 year, walking (or exercise equal to it) for ≥1 hour a day, walking faster than other people of the same age and sex.	Rome IV criteria	The rate of functional constipation was significantly lower in people who exercised at least twice a week for at least one year for at least 30 minutes.
10	Yamada et al.	Lifestyle, psychological stress, and incidence of adolescent constipation: results from the Toyama birth cohort study	2021	Japan	5,540 adolescents aged 12 to 13	Prospective study	Physical activity (“active” or “inactive”) and watching TV on weekdays (< 2, < 3, or ≥ 3hours).	The answer was classified into three categories: at least once daily, once every 2 days, or less frequently than once every 2 days. Then, bowel movements less frequently than once every 2 days were defined as constipation because this corresponds to the Rome IV criterion of “two or fewer defecations in the toilet per week"	Being physically inactive was significantly associated with the incidence of constipation.
11	Harada et al.	Associations between lifestyle factors and constipation among survivors after the Great East Japan earthquake: a 9-year follow-up study	2023	Japan	9,234 survivors aged 18 years or older	Cross-sectional survey	Good (≥23 METs hours/week), poor (<23 METs hours/week).	Present or absent	In both men and women, physical activity was significantly associated with higher odds ratios of constipation.
12	Tuteja et al.	Is constipation associated with decreased physical activity in normally active subjects?	2005	United States	A total of 1,069 employees (age range 24–77)	Cross-sectional survey	Kaiser Physical Activity Survey (KPAS)	Rome I criteria	The average total physical activity and all subscales of physical activity were not significantly different in subjects with and without constipation (all p-values≥0.2).
13	Derbyshire et al.	Diet, physical inactivity and the prevalence of constipation throughout and after pregnancy	2006	United Kingdom	94 primiparous non-smokers aged 19–40 years (mean: 33.4 years)	Case-control study	Vigorous activities referred to activities that took hard physical effort, causing volunteers to breathe much harder than normal (fast bicycling, swimming, and aerobics). Moderate activities referred to activities taking moderate physical effort and made volunteers breathe somewhat harder than normal (bicycling and swimming at a regular pace). Light activities were defined as those requiring minimal physical exertion (walking and meditating).	Rome II diagnostic criteria	No statistically significant differences were identified between light, moderate, and vigorous physical activity levels when groups were compared.
14	López Cara et al.	Constipation in the population over 50 years of age in Albacete province	2006	Spain	414 participants over 50 years of age	Cross-sectional survey	The participants were divided into two groups based on whether or not they habitually exercised on a daily basis.	Participants who had bowel movements less than three times a week were considered to have constipation.	Student's t-test did not find statistically significant differences (t = -0.831, p = 0.407)
15	Santacruz et al.	Do bad habits bring a double constipation risk?	2018	Spain	415 of 910 eligible subjects were randomly selected. The mean subject age was 43.8±11.9 years. Of the subjects, 181 were medical staff members, 78 were nurses, and 64 were auxiliary nurses, together reaching 77.8% of the recruited population.	Cross-sectional survey	Definitions of exercise were provided, considering regular exercise to be three or more days a week of any type of aerobic exercise (walking/running, cycling, swimming, or other).	Rome III criteria and subjects were asked whether they felt constipated.	Only 45.1% of the subjects in the present population reported regular exercise, but no association of this habit with constipation was observed.
